# *Clostridium* associated emphysematous gastritis: A case report and review of literature^1^

**DOI:** 10.1016/j.idcr.2026.e02507

**Published:** 2026-01-25

**Authors:** Roma Tarar, Chinedu Ezekekwu, Chibuike Enwereuzo, Anishur Rahman

**Affiliations:** a New York Institute of Technology College of Osteopathic Medicine, 100 Northern Blvd Glen Head, NY 11542, USA; b UHS Wilson Medical Center, 33-57 Harrison St., Johnson City, NY 13790, USA.

**Keywords:** Emphysematous gastritis, *Clostridium perfringens*, Necrotizing infection, Gastric necrosis, Gas gangrene, Abdominal surgery

## Abstract

Emphysematous gastritis is a rare, often fatal infection of the stomach wall characterized by gas formation and tissue necrosis, most frequently associated with polymicrobial infections. Cases with demonstration of tissue invasion by *Clostridium* species are exceptionally uncommon. We report the case of a 77-year-old woman with multiple comorbidities, including diabetes mellitus, obesity, atherosclerosis, and chronic kidney disease, who presented with severe abdominal pain, nausea, and vomiting. Imaging revealed gastric emphysema with pneumoperitoneum. Emergent laparotomy demonstrated full-thickness necrosis of the stomach along the lesser curvature, necessitating total gastrectomy with Roux-en-Y esophagojejunostomy and splenectomy. Histopathology confirmed extensive necrosis with gas dissection and abundant gram-positive, boxcar-shaped bacilli consistent with *Clostridium perfringens*. This case highlights the pathogenic potential of *C. perfringens* to cause spontaneous, necrotizing emphysematous gastritis in the absence of trauma or malignancy. Vascular compromise from severe atherosclerosis and thrombosis likely contributed to tissue hypoxia, creating a permissive environment for clostridial invasion. A literature review identified only a handful of human cases of emphysematous gastritis with definitive *Clostridium* identification, most with fatal outcomes unless surgically managed.

**Conclusion:**

Emphysematous gastritis associated with *Clostridium* species is exceedingly rare and rapidly progressive. Early recognition and aggressive surgical resection appear to offer the best chance of survival. Clinicians should maintain a high index of suspicion in high-risk patients presenting with acute abdominal symptoms and gastric wall emphysema.

## Highlights

• Exceptionally rare emphysematous gastritis with *Clostridium* associated invasion.
• Survival achieved after emergent total gastrectomy with Roux-en-Y reconstruction.
• Highlights importance of early diagnosis and treatment in high-risk cases.

## Introduction

The Clostridia are anaerobic, spore-forming bacilli that are part of the normal intestinal flora of man and animals. They usually cause disease when abnormal tissue provide a relatively anaerobic environment that gives the bacilli a selective growth advantage, favor toxin production and disease establishment [1]. Clostridia associated with human diseases are gram positive [1]. Clostridial myonecrosis or gas gangrene, most frequently caused by *C. perfringens* is a severe, often-fatal disease characterized by local tissue necrosis, systemic toxemia and gas production from anerobic fermentation [1], [2]. Most cases of intra-abdominal gas gangrene are usually preceded by trauma or surgery, seeding previously sterile tissue with clostridium spores or bacilli [1], [2]. Malignant colorectal neoplasms or some underlying diseases are often seen in cases not previously associated with trauma [2], [3]. Emphysematous gastritis is a rare, life threatening manifestation of Clostridium, characterized by the presence of gas within the gastric wall. [4], [5] The condition has been associated with alcoholism, diabetes mellitus, renal failure, recent abdominal surgery, gastroenteritis, long-term corticosteroid use, ingestion of corrosive agents, and certain medication. [5], [6] *Streptococcus* species*, Escherichia coli, Enterobacter* species*, Clostridium* species*, Pseudomonas aeruginosa, Staphylococcus aureus, Candida* species*,* and *Mucor* species have all been implicated in the etiology and the infection has often been reported as polymicrobial. [4], [5], [6] Spontaneous cases, confined to the stomach and not previously associated with trauma, bowel neoplasm or disease have only rarely been reported. We present a case of necrotizing, emphysematous gastritis, associated with prominent tissue invasion by *Clostridium Spp.*

## Case summary

The patient is a 77 year-old woman who presented with a five-day history of upper abdominal pain radiating to the back, accompanied by nausea and vomiting. There was no history of melena, hematemesis, or prior esophagogastroduodenoscopy. The patient's past medical history was notable for morbid obesity, type 2 diabetes mellitus, hypertension, coronary artery disease, stage 3 chronic kidney disease, obstructive sleep apnea, chronic obstructive pulmonary disease, and a history of nicotine abuse. On presentation, the patient was febrile and tachycardic, with laboratory findings significant for leukocytosis, elevated serum lactate, procalcitonin, and C-reactive protein levels, consistent with a systemic inflammatory response.

**Fig. 1:**
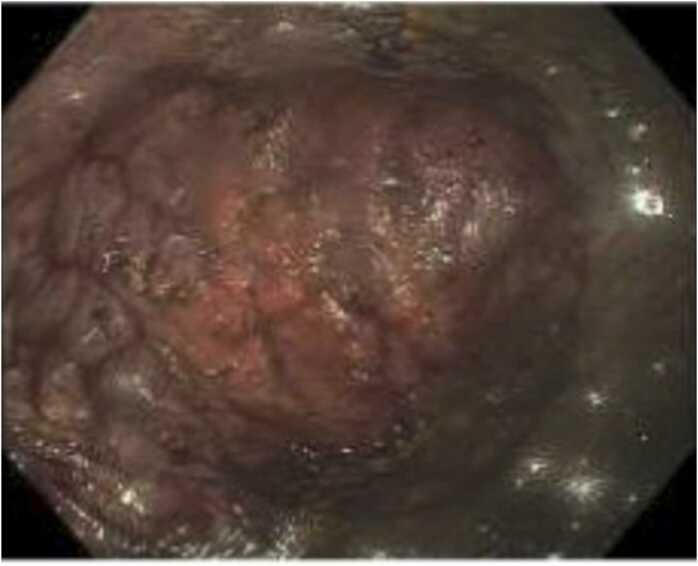
Gastric endoscopy, showing gastric necrosis.

A non-contrast computed tomography (CT) scan of the abdomen revealed pneumoperitoneum and extensive gastric emphysema. Additionally, marked aortic atherosclerosis and a dilated descending aorta were observed. The patient underwent emergency surgery the same day, including exploratory laparotomy, total gastrectomy and Roux-en-Y esophagojejunostomy. A splenectomy was performed due to active splenic hemorrhage. Intraoperative inspection revealed full-thickness necrosis along the entire length of the lesser curvature of the stomach, extending from the gastroesophageal (GE) junction to the prepyloric region. An intraoperative upper gastrointestinal endoscopy confirmed ischemic changes extending beyond the GE junction into the stomach, while the esophagus appeared uninvolved (see Fig. 1). The stomach was resected, together with a portion of the duodenum and spleen and submitted for pathological evaluation.

## Pathology

**Fig. 2:**
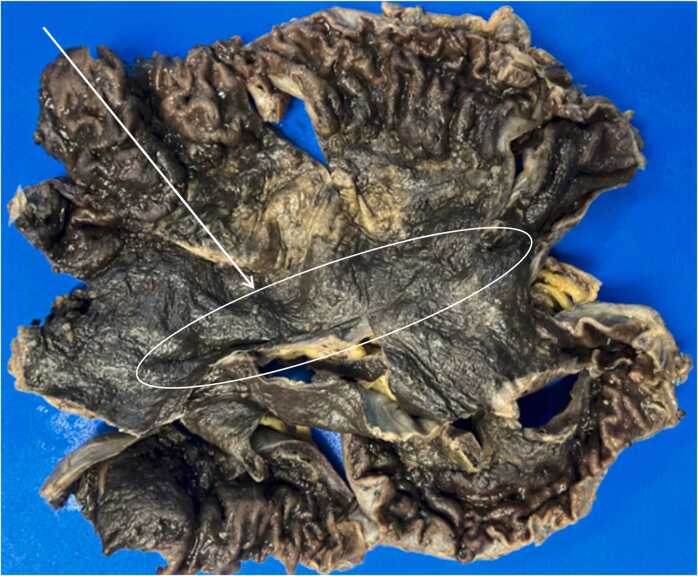
Formalin fixed tissue showing loss of gastric folds along the lesser curvature (white arrow and circle) with relative sparing of the greater curvature.

**Fig. 3:**
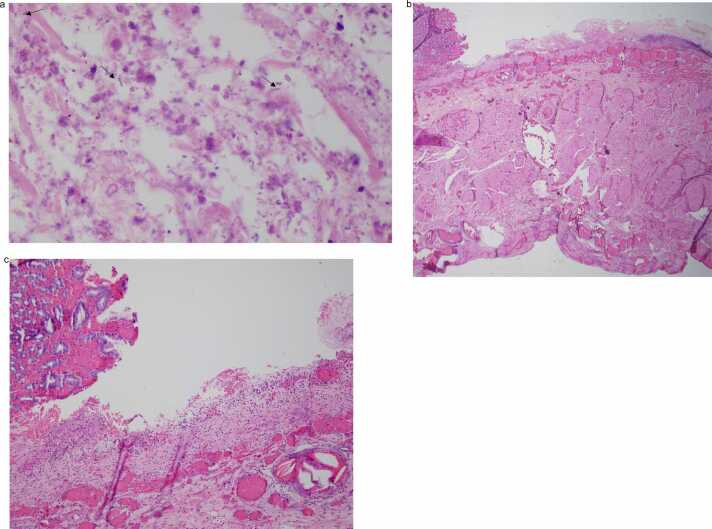
a. H&E section of gastric tissue showing transmural necrosis, necro-inflammatory debris and gas expanding and dissecting through tissue planes. The black arrows show numerous plump bacilli visible in the section (x60 objective). Fig. 3b. Low power view of the stomach cross section showing extensive mucosal necrosis and hemorrhage (x2 objective). Fig. 3c. High power view of the stomach cross section showing mucosal necrosis, partially necrotic and hemorrhagic (x10 objective).

**Fig. 4:**
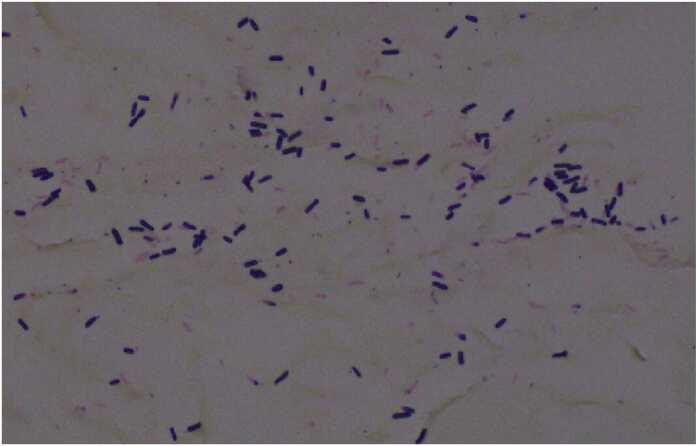
Gram stain confirms numerous, plump, “boxcar” shaped gram-positive organisms, morphologically consistent with *Clostridium Spp.*

On gross examination, the gastric serosa was greyish brown and appeared necrotic with thinned areas along the lesser curvature (see Fig. 2). The mucosa surface was erythematous, with gray-brown, flattened areas and loss of gastric folds, corresponding to the necrotic areas noted on the serosa (see Fig. 2). Microscopy revealed extensive necrosis with gas dissection into tissue planes. Prominent, plump boxcar-shaped bacilli were present throughout the necrotic tissue (see Fig. 3a-c). These bacilli were demonstrated to be gram positive on gram stain and morphologically consistent with *Clostridium perfringens* (see Fig. 4). No notable pathological alterations were identified in the resected portion of the duodenum. The spleen exhibited patchy areas of necrosis, subcapsular hemorrhage, as well as multifocal, calcifying vascular thrombosis.

## Discussion

This patient presented with necrotizing emphysematous gastritis, localized along the lesser curvature, which corresponds to the distribution of the left gastric artery. The stomach typically has a rich vascular network, receiving arterial blood from multiple primary sources with collateral circulation that protects the stomach from ischemic injury, making isolated gastric necrosis an exceptionally rare event. Clostridium rarely invades healthy tissue and requires a localized reduction in oxygen tension to establish disease. The patient’s history of type II diabetes mellitus, atherosclerotic disease and morbid obesity (BMI-49) increases the risk of vascular disease. A localized thrombosis may have caused tissue hypoxia, providing a nidus for Clostridium to invade, multiple and amplify the necrosis. This possibility is supported by involvement of a vascular distribution area (the lesser curvature perfused by the left gastric artery) and partial viability around the greater curvature, as well as the patchy vascular thrombosis present in the spleen.

Gangrenous tissue infection caused by *Clostridium* spp, especially *C. Perfringens* is a frequently fatal condition that requires prompt surgical resection of affected tissue to control the infection and offer any chance of survival. Our review of literature showed about four cases of emphysematous gastritis in humans with definitive identification of Clostridium species in clinical specimens and two cases in veterinary medicine. Of the four cases reported in humans, only one was caused by *Clostridium perfringens.*[7] The patient had gastric wall emphysema and the disease had a fulminant course that was rapidly fatal. *Clostridium perfringens* was identified in multiple organs on post mortem microbial culture. The remaining 3 cases reported in human were caused by *Clostridium ventriculi.*[8], [9], [10] Of the three reported cases attributed to *Clostridium ventriculi*, two did not receive surgical intervention and resulted in fatality. [9], [10] The last case caused by *Clostridium ventriculi* responded to gastrectomy with full recovery. [8], [9] Two of the cases caused by *Clostridium ventriculi* occurred in patients with weakened immunity from cancer chemotherapy and immunosuppressant for organ transplant, respectively. This suggests immunosuppression may play some role in disease pathogenesis for cases caused by *Clostridium ventriculi.* At least 2 fatal cases have been reported in veterinary medicine, due to *Clostridium perfringens* and *Clostridium septicum* involving a cat and a horse respectively. [11], [12] Table 1 shows a list of emphysematous gastritis with demonstration of clostridium in clinical specimen.

**Table 1:** A summary of of reported cases of emphysematous gastritis. The index case is number 1.

	Specie	Age	sex	Organism	Co-morbidity	Treatment	Outcome
1.	Human	77 y	F	*Clostridium perfringens*	DM II, Hypertension, Obesity, Atherosclerosis	Gastrectomy	Recovery
2.	Human	76 y	F	*Clostridium perfringens*	Hypertension, Obesity, Atherosclerosis	Medical	Fatal [7]
3.	Human	80 y	F	*Clostridium ventriculi*	Breast cancer on Paclitazel chemotherapy	Gastrectomy	Recovery [8]
4.	Human	35 y	M	*Clostridium ventriculi*	DM, Kidney transplant on immunosupressant	Medical	Fatal [9]
5.	Human	86 y	F	*Clostridium ventriculi*	DM II, Hypertension	Medical	Fatal [10]
6.	Cat	10	M	*Clostridium perfringens*	Neutered	Medical	Fatal [11]
7.	Horse	3.5 y	M	*Clostridium septicum*		Exploratory celiotomy	Euthanized [12]

We recognize that conclusive attribution of the disease process to *Clostridium perfringens* cannot be established based exclusively on histomorphologic features, as other clostridial species may exhibit overlapping characteristics. Molecular confirmation, such as PCR-based detection of *C. perfringens* toxin genes from formalin-fixed paraffin-embedded tissue, would provide greater evidence of species level identification and toxin profiling, as described by Rood et al. [13] Unfortunately, bacteriologic culture was not performed at the time of surgery, and subsequent efforts to obtain molecular confirmation through multiple commercial reference laboratories were unsuccessful, as such testing is not currently available. Despite this limitation, the observed histopathologic features, namely large, boxcar-shaped, gram-positive bacilli in association with extensive tissue necrosis and gas formation, are most consistent with *C. perfringens* and do not correspond to morphology typically described for clostridial species known to cause human disease. Furthermore, the fulminant clinical course and necrotizing emphysematous gastritis observed in this case align with previously reported manifestations of *C. perfringens,* associated infection. Tissue blocks from this case remain available, and we would be willing to pursue PCR-based toxin gene analysis should be an appropriate reference laboratory with expertise in testing formalin-fixed specimens be identified. While conclusive strain typing is not possible under the present circumstances, the convergence of clinical, histopathologic and epidemiologic features supports *C. perfringens* as the most likely etiologic agent.

## Conclusion

In conclusion, the identification of *clostridium species* in the clinical specimens of patients with emphysematous gastritis or spontaneous gastric necrosis could be alarming and life threatening if not quickly identified and treated. The precise trigger or cause of the condition and why the disease is often confined to the stomach is still unclear. A high index of suspicion is necessary for prompt diagnosis. From the reported cases, gastric resection is the most promising intervention that offers the best chance of survival.

## Author agreement statement

We the undersigned declare that this manuscript is original, has not been published before and is not currently being considered for publication elsewhere. We confirm that the manuscript has been read and approved by all named authors and that there are no other persons who satisfied the criteria for authorship but are not listed. We further confirm that the order of authors listed in the manuscript has been approved by all of us. We understand that the Corresponding Author is the sole contact for the Editorial process. She is responsible for communicating with the other authors about progress, submissions of revisions and final approval of proofs.

## CRediT authorship contribution statement

**Chibuike Enwereuzo:** Writing – review & editing, Writing – original draft, Data curation, Conceptualization. **Anishur Rahman:** Supervision. **Chinedu Ezekekwu:** Writing – review & editing, Writing – original draft, Data curation, Conceptualization. **Roma Tarar:** Writing – review & editing, Writing – original draft, Data curation, Conceptualization.

## Consent

Written informed consent was obtained from the patient for publication of this case report and accompanying images. A copy of the written consent is available for review by the Editor-in-Chief of this journal on request.

## Patient consent

None

## Ethical Approval

This study did not involve human participants or animals, therefore IRB/ethical approval was not required.

## Funding

This research received no external funding.

## Declaration of Competing Interest

All authors declare that there is no conflict of interest regarding the publication of this paper.
